# Cannabis and cocaine decrease cognitive impulse control and functional corticostriatal connectivity in drug users with low activity DBH genotypes

**DOI:** 10.1007/s11682-015-9488-z

**Published:** 2015-12-14

**Authors:** J. G. Ramaekers, J. H. van Wel, D. Spronk, B. Franke, G. Kenis, S. W. Toennes, K. P. C. Kuypers, E. L. Theunissen, P. Stiers, R. J. Verkes

**Affiliations:** 1 Department of Neuropsychology and Psychopharmacology, Faculty of Psychology and Neuroscience, Maastricht University, Maastricht, The Netherlands; 2 Department of Psychiatry, Donders Institute for Brain, Cognition and Behaviour, Radboud University Medical Center, Nijmegen, The Netherlands; 3 The Netherlands Forensic Psychiatric Centre Pompestichting, Nijmegen, The Netherlands; 4 Department of Human Genetics, Radboud University Medical Center, Nijmegen, The Netherlands; 5 Department of Psychiatry and Neuropsychology, School for Mental Health and Neuroscience, Maastricht University, Maastricht, The Netherlands; 6 Department of Forensic Toxicology, Institute of Legal Medicine, Goethe University of Frankfurt, Frankfurt, Germany

**Keywords:** Impulse control, Cannabis, Cocaine, DBH genotype, Fuctional connectivity

## Abstract

The dopamine β-hydroxylase (DβH) enzyme transforms dopamine into noradrenaline. We hypothesized that individuals with low activity DBH genotypes (rs1611115 CT/TT) are more sensitive to the influence of cannabis and cocaine on cognitive impulse control and functional connectivity in the limbic ‘reward’ circuit because they experience a drug induced hyperdopaminergic state compared to individuals with high activity DBH genotypes (rs1611115 CC). Regular drug users (*N* = 122) received acute doses of cannabis (450 μg/kg THC), cocaine HCl 300 mg and placebo. Cognitive impulse control was assessed by means of the Matching Familiar Figures Test (MFFT). Resting state fMRI was measured in a subset of participants to determine functional connectivity between the nucleus accumbens (NAc) and (sub)cortical areas. The influence of cannabis and cocaine on impulsivity and functional connectivity significantly interacted with DBH genotype. Both drugs increased cognitive impulsivity in participants with CT/TT genotypes but not in CC participants. Both drugs also reduced functional connectivity between the NAc and the limbic lobe, prefrontal cortex, striatum and thalamus and primarily in individuals with CT/TT genotypes. Correlational analysis indicated a significant negative association between cognitive impulsivity and functional connectivity in subcortical areas of the brain. It is concluded that interference of cannabis and cocaine with cognitive impulse control and functional corticostriatal connectivity depends on DBH genotype. The present data provide a neural substrate and behavioral mechanism by which drug users can progress to drug seeking and may also offer a rationale for targeted pharmacotherapy in chronic drug users with high risk DBH genotypes.

## Introduction

Reward seeking and impulsive behaviors such as addiction have been associated with dopamine transmission in the limbic ‘reward’ circuitry (Pierce and Kumaresan 2006; Volkow et al. 2007). The latter constitutes a cortico-subcortical network that receives modulatory input to the nucleus accumbens (NAc) from ascending dopaminergic projections from the ventral tegmental area (Alexander et al. 1986; Bonelli and Cummings 2007; Perreault et al. 2011). The circuit broadly consists of a reflective system located in the medial prefrontal cortex and a reactive system located in the striatum. The reactive system is sensitive to reward and generates impulses following dopaminergic stimulation of the NAc whereas the reflective system operates glutamatergic control over impulses generated in the striatum (Bechara 2005; Faure et al. 2010; Kalivas and Volkow 2005). All drugs of abuse increase dopamine transmission in the NAc (Nestler 2005) which is essential for acute drug reward (Dagher and Robbins 2009; Kalivas and Volkow 2011; Volkow et al. 2011) and may be very relevant for progressively shaping drug use into drug seeking behavior (Kalivas 2009) by reducing inhibitory control over the reactive system (D’Amour-Horvat and Leyton 2014).

Vulnerability to drug addiction and loss of inhibitory control may be affected by inter-individual variations in genetic expression and tonic dopamine levels in the limbic circuit (Koob 1998; Kreek et al. 2005; Volkow et al. 2007, 2011). It has been posed that the relationship between tonic dopamine and performance is non-linear and inverted-U shaped, with both excessive as well as insufficient levels of dopamine impairing cognitive control (Cools 2011; Cools and D’Esposito 2011). A prime factor influencing tonic dopamine in the brain is the enzyme dopamine beta-hydroxylase (DβH) (Hess et al. 2009; Kreek et al. 2005; Weinshenker and Schroeder 2007) that catalyzes the biotransformation of dopamine into noradrenaline. A polymorphism in the DBH encoding gene, the single nucleotide polymorphism rs 1611115 (−970C-T, formerly known as -1021C > T) accounts for up to 50 % of the variance in the DβH enzyme activity (Zabetian et al. 2001). Individuals with the high activity CC genotype are expected to display low levels of dopamine. Individuals with low activity genotypes (CT and TT) are expected to display high levels of dopamine and have been associated with impulsive personality styles (Hess et al. 2009). Thus, tonic dopamine levels in the limbic circuit of drug users and their response to drugs of abuse may vary considerably between individuals depending on their genetic make-up at the DBH locus.

The present study was designed to measure whether inter-individual differences in tonic dopamine metabolism (i.e. caused by DBH polymorphism) modulate acute effects of cannabis and cocaine on cognitive impulse control and functional connectivity between striatal and cortical parts of the limbic reward circuit. Cognitive impulsivity was assessed by means of the Matching Familiar Figures Test (MFFT). The latter has previously been used to demonstrate loss of cognitive impulse control in drug users (Morgan 1999; Quednow et al. 2007). Resting state fMRI was measured to determine functional connectivity between the regions of interest (ROI) in the NAc and remote cortical areas (Ramaekers et al. 2013). DBH genotype was determined in all participants. It was expected that individuals with high tonic levels of dopamine (i.e. carriers of CT/TT genotypes) would be more sensitive to the impairing potential of cannabis and cocaine on cognitive impulse control because they would experience a drug induced hyperdopaminergic state. Likewise, it was expected that dopaminergic stimulation of the NAc following both drugs would reduce functional corticostriatal connectivity (Ramaekers et al. 2013), and more so in CT/TT individuals.

## Methods

### Participants

Participants were recruited at 2 study sites (i.e. Maastricht and Nijmegen) that participated in this multicenter trial. In total, 122 regular users of cannabis and cocaine (male *N* = 96, female *N* = 26) entered the study. On average, participants had been regular users of cannabis and cocaine for 7 yrs. (min-max: 1–23) and 3.2 yrs. (min-max:0.5–6) respectively. Participants reported an average (SD; min-max) use of cannabis and cocaine on 44.8 (26.8) and 3.7 (3.5) occasions respectively during the previous 3 months. Participants also reported the use of other substances such as MDMA (88 %), amphetamines (73 %), mushrooms (61 %), LSD (20 %) and a range of miscellaneous drugs (60 %) such as nitrous oxide, MDMA crystals, DMT and ketamine. Mean (SD) age of participants was 22.8 (3.7) yrs. Mean (SD) trait impulsivity score as assessed with the Barratt Impulsiveness Scale was 69.5 (9.6).

Participants were recruited through advertisements in local newspapers, flyers distributed at targeted locations (e.g., college campuses, bars, night clubs, concerts, head shops) and by word of mouth. Candidates received a medical examination by the medical supervisors who determined study eligibility. The medical supervisor checked vital signs, conducted a resting 12-lead electrocardiogram (ECG), took blood and urine samples. Participants filled out a standard questionnaire on medical history. Standard blood chemistry, hematology, DBH genotyping and drug screen tests were conducted on blood and urine samples respectively.

Inclusion criteria were: written informed consent; age 18–40 yrs.; (regular) use of cannabis (i.e. ≥ 2 times/3 mo); cocaine use at least 5 times in the previous year, good physical and mental health and normal weight (BMI 18-28). Exclusion criteria were: cocaine dependence according to DSM-IV criteria; use of psychotropic medicinal drugs, presence or history of psychiatric or neurological disorder; pregnancy or lactating; cardiovascular abnormalities; excessive alcohol use (>20 units/week) or smoking (>15 cigarettes/day), and hypertension.

This study (Dutch Trial Register, trial number NTR2127) was conducted according to the code of ethics on human experimentation as described in the declaration of Helsinki (1964), amended in Seoul (2008). The study protocol was approved by the Medical Ethics Committee of Maastricht University.

### Design, drug dose and administration

Participants entered a double-blind, placebo-controlled, 3-way crossover study. Treatments consisted of placebo, 450 μg/kg THC (cannabis plant material, divided over 2 successive doses of 300 and 150 μg/kg) and a single dose of 300 mg cocaine HCl. Cannabis was prepared from batches that contained 11–12 % THC. Cannabis doses were tailored to each individual subject to represent weight calibrated doses of 300 μg/kg THC and 150 μg/kg THC and administered using a Volcano vaporizer (Storz & Bickel Volcano ®). Cocaine HCl and placebo were administered in an opaque white capsule. Treatments were administered using a double dummy technique to synchronize time of maximal drug concentrations (Tmax) during performance testing and resting state fMRI. Cocaine or cocaine placebo capsules were administered at 1,25 h prior (T0) to assessment of performance in the MFFT, whereas the first dose of cannabis (300 μg/kg THC) or cannabis placebo was inhaled 15 (T1) min prior to MFFT. A second dose (T2) of cannabis (150 μg/kg THC) or cannabis placebo was administered one hour after the first dose (T1) and 30 min prior to resting state fMRI. MFFT performance was collected from participants in both centers. However only participants recruited by the Maastricht center (N = 61) received a subsequent fMRI session. Treatment conditions were separated by a minimum wash-out period of 7 days and counterbalanced across participants. Vital signs and blood samples were taken after treatment administrations (T0, T1 and T2).

### Procedures

Participants were familiarized with tests and procedures on a separate training day prior to the treatment conditions. Participants were instructed to refrain from drug use (except for cannabis) throughout study participation. In the morning of test days, absence of benzodiazepines, opiates, cocaine, marijuana, MDMA and (meth)amphetamine was assessed using urine screens and the absence of alcohol was measured using a breathalyzer. Female participants underwent an additional pregnancy test. Participants were only allowed to proceed when test results for drug (except cannabis) and alcohol use and pregnancy were negative. Participants that passed the screen test received a standard breakfast, followed by baseline measures of vital signs (blood pressure and heart rate) and a sample of blood.

### Matching familiar figures test (MFFT)

The MFFT measures cognitive impulsivity. It involves simultaneous presentation of a target figure centered on the left half of the screen and an array of six alternatives on the right half of the screen, all except one differing in one or more details from the target figure. The participants are asked to find the item that exactly matches the target item by pressing a keyboard number corresponding to each of the items. If the initial selection is incorrect, this is signaled with a beep and participants are requested to respond again. Each subject is given 2 practice trials followed by 20 test trials. Mean latency of the first response and total number of errors, are recorded and used to calculate two primary measures after z-transformation : i.e. an Impulsivity score (I-score) and an Efficiency score (E-score) (Messer and Brodzinsky 1979). The I-score is a composite index of impulsivity and is calculated by subtracting the standardized score of the mean latency to first response from the standardized score of the total number of errors committed (Zerror-Zlatency). The E-score is calculated by summing the standardized score of the mean latency to first response from the standardized score of the total number of errors committed (1-(Zerror + Zlatency)).

### Resting state fMRI

MRI images were obtained in a 3 T head-only scanner (Siemens® MAGNETOM Allegra). During resting state 240 EPI whole brain functional volumes were acquired (TR = 2 s, TE = 30 ms, flip angle = 90°, matrix = 64 × 64 × 32, voxel size = 3.5 × 3.5 × 3.5 mm). Volunteers were asked to relax with the eyes open during resting state measurement. For anatomical reference, a 3D MPRAGE (magnetization-prepared rapid gradient echo; TR = 9.7 ms, TE = 4 ms, flip angle = 12°, matrix = 256 × 256, voxel size = 1 × 1 × 1 mm^3^) image data set was acquired.

The fMRI data were preprocessed using SPM8 software (Welcome Trust Center for Neuroimaging, London, UK). The first 2 volumes were removed from each fMRI data set to allow for magnetic equilibration. Preprocessing included 3D motion correction and slice time correction. Individual anatomical data sets were normalized to standard 3-D MNI space. Individual functional images were realigned, co-registered and normalized to the anatomical data, and resampled to a voxel size of 3 × 3 × 3 mm^3^. Spatial smoothing was conducted with a FWHM 4 mm Gaussian kernel.

### Functional connectivity

Functional connectivity data were produced with the MATLAB toolbox DPARSFA (Chao-Gan and Yu-Feng 2010). First, linear trends of time courses were removed followed by low band-pass filtering (0.01–0.08 Hz) of the preprocessed data to remove ‘noise’ attributable to physiological parameters. In addition, nuisance covariates including 6 motion parameters, the white matter signal and the CSF signal were removed. Two spheres (radius 4 mm) were created that were located (in MNI space) in the NAc of the left (−9, 9,-9) and right (9, 9,-9) hemisphere (Di Martino et al. 2008). Average time courses were obtained for each sphere separately and correlational analysis was performed voxel wise to generate functional connectivity maps for each sphere. Finally, the correlation coefficient map was converted into z-maps by Fisher’s r-to-z transform to improve normality. Drug-induced changes in functional connectivity of the NAc have been shown to serve as a useful marker of drug activity in in the brain reward circuit (Ramaekers et al. 2013).

### Pharmacokinetics and pharmacogenetics

Serum was used for detection of cannabinoids whereas cocaine and metabolites were determined in plasma. The determination of Δ9-tetrahydrocannabinol (THC), 11-hydroxy-THC (THC-OH), 11-nor-9-carboxy-THC (THC-COOH), cocaine (COC), benzoylecgonine (BZE) and ecgonine methyl ester (EME) in plasma was performed in a specialized forensic-toxicological laboratory using validated procedures 38,39. DBH genotype was analyzed after DNA isolation from blood collected at medical screening using standard protocols. The DBH polymorphism (rs1611115) was genotyped using restriction fragment length polymorphism (RFLP) analysis. PCR was performed on 50 ng genomic DNA using 0.2 μM forward primer (5’-TGAATGTGCCCCTAAGGCTA-3’) and 0.2 μM reverse primer (5’-CACCTCTCCCTCCTGTCCTCTCGC-3’), 0.25 mM dNTPs, 2 U Taq DNA polymerase (Invitrogen, Breda, The Netherlands) in a 1× PCR optimization buffer A (30 mM Tris-HCl pH 8.5, 7.5 mM (NH4)2SO4, 0.75 mM MgCl2). The cycling conditions were 5 min 92 °C, followed by 35 cycles of 1 min 92 °C, 1 min 59 °C, 1 min 72 °C., and a subsequent 5 min 72 °C.To purify the PCR-products, NucleoFast96 plates (Macherey-Nagel, Düren, Germany) were used. Restriction reaction was performed in a total volume of 15 μl, containing 10 μl purified PCR-product, 5 U HhaI, 1× NEBuffer 4 and 1× BSA. Digestion was performed at 37 °C for 16 h, which resulted in two fragments of 200 bp and 24 bp for the C allele, the T variant remained uncut (224 bp).

### Statistics

A total of 122 participants entered the study. DBH genotype data was missing in 6 participants and MFFT datasets of another 6 participants were incomplete, due to technical failures. Consequently, 110 participants with complete datasets entered the statistical analyses of MFFT data. For analysis, individuals with low activity DBH genotypes, i.e. CT (*N* = 33) and TT (*N* = 4), were pooled (CT/TT) and compared to CC homozygotes (*N* = 73). A total of 61 participants (the Maastricht sample) participated in resting state measures. fMRI datasets were incomplete for 22 participants due to technical failures or due to excessive movement during drug conditions, rendering 39 participants with complete fMRI datasets. DBH genotype was missing in 4 of those participants, which rendered 35 participants with complete datasets to test for Drug x DBH interactions on functional connectivity.

Effects on impulse control tasks from oral THC and cocaine HCl in dosages similar to those proposed for this study have achieved statistical significance in within subject designs employing 20 or less participants (Fillmore et al. 2006; Ramaekers et al. 2006a; Ramaekers and Kuypers 2006). Assuming an omnibus *p* < 0.05 and power = 0.8, we estimated that the present study employing 122 subjects would enable detection of performance differences between drug treatments with an effect size of 0.3 (i.e., a signal change of .3 times the standard deviation) in within subject comparisons. In addition, we estimated that between DBH group comparisons of genotypes would enable detection of performance differences with an effect size of .7 and .4 for sizes of *N* = 30 and *N* = 60 respectively. Straightforward power analysis for imaging data is less feasible. Because fMRI data analysis consists of a very large number of non-independent multiple comparisons, a proper power analysis with correction for multiple comparisons requires computer simulation. In addition, statistical power is not the same (i.e., homogenous) over the brain, but varies across brain regions (Petersson et al. 1999). A common approach in the functional neuroimaging community is to estimate the number of subjects from previous empirical experience in the field, particularly when pilot data are lacking and analyses include the whole brain or multiple regions of interest (Mumford 2012). Previous resting state fMRI studies have employed 15–20 subjects for showing significant drug effects (Honey et al. 2003; Kelly et al. 2009; Ramaekers et al. 2013; Schrantee et al. 2015). Based on these experiences we expected no problem to demonstrate drug effects on fMRI measures in 60 participants and that sample sizes of subgroups DBH genotypes (*N* = 15–30) would be sufficient for demonstrating between group differences.

MFFT measures were analyzed with SPSS 21.0 using a GLM univariate analysis of variance (ANOVA) with Drug (3 levels; placebo, cannabis and cocaine) as within subject factor and DBH genotype (2 levels; CC and CT/TT) as between group factor. The model tested for main effects of Drug, DBH genotype and their interaction. Significant main effects were subsequently followed by drug-placebo contrasts to establish the separate effects of cannabis (2 within subject levels; placebo and cannabis) and cocaine (2 within subject levels; placebo and cocaine) and their interaction with DBH genotype (2 between group levels; CC and CT/TT).

Functional connectivity data (i.e. correlation coefficient maps for each individual in each treatment condition) were analysed in 2 GLM models of ANOVA in SPM8. In the first GLM, complete datasets of 39 participants were analysed using ANOVA with Drug (3 levels) as within subject factor. The GLM model established the effects of cannabis and cocaine separately by means of drug-placebo contrasts. DBH genotyping was only available for 35 participants (CC genotype *N* = 22; CT/TT genotype *N* = 13) that were included in GLM 1. Therefore a 2nd GLM was conducted that included 35 participants for which both resting state and DBH data sets were complete. These data were entered in an ANOVA with Drug (3 levels) as within subject factor and DBH genotype (2 levels) as between group factor. From this model, main effects of DBH genotype and its interaction with cannabis and cocaine treatment were identified. The statistical threshold was set at *p* < .05 FWE cluster corrected (cluster size >50).

Finally, voxel wise correlation analysis between FC data and MFFT composite measures of Impulsivity and Efficiency were conducted.

## Results

### Matching familiar figures test (MFFT)

All MFFT measures demonstrated a significant main effect of the factor Drug. The interaction factor Drug x DBH genotype also reached significance for all MFFT measures except Efficiency. There was no main effect of DBH genotype in any of the MFFT parameters.

**Fig. 1:**
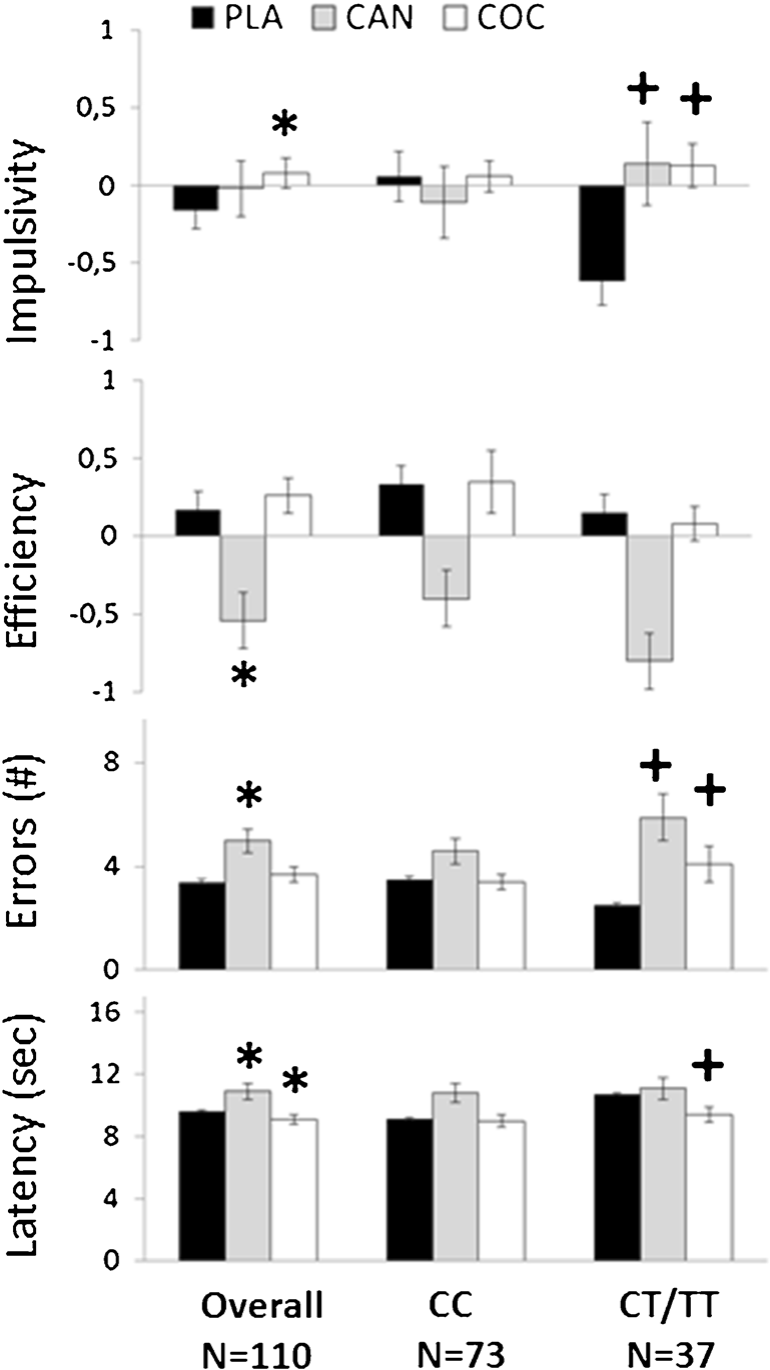
Mean (se) performance in the MFFT during drug and placebo challenges averaged over DBH groups and for carriers of CC and CT/TT genotypes separately (CAN = cannabis, COC = cocaine, PLA = placebo); * = *p* < 0.05 (drug-placebo contrasts across genotypes); + = *p* < 0.05 (drug x DBH genotype interaction)

Separate contrasts revealed that cocaine significantly increased Impulsivity (F_1,108_ = 8,9; *p* = .004) and decreased Response latency (F_1,108_ = 7.0; *p* = .009) overall. Cannabis significantly reduced Efficiency (F_1,108_ = 27.6; *p* = .000), increased Errors (F_1,108_ = 20.5; *p* = .000) and Response latency (F_1,108_ = 5.2; *p* = .024) overall. The influence of cocaine on MFFT performance significantly interacted with DBH genotype. Cocaine increased Impulsivity (F_1,108_ = 9.0; *p* = .003) and Errors (F_1,108_ = 4.0; *p* = .049) and decreased Response latency (F_1,108_ = 6.1; *p* = .015) in CT/TT individuals but not in CC individuals. The influence of cannabis on MFFT performance also significantly interacted with DBH genotype. Cannabis increased Impulsivity (F_1,108_ = 6.2; *p* = .014) and Errors (F_1,108_ = 5.5; *p* = .021) in CT/TT but not in CC individuals. Mean (se) performance in the MFFT in each drug condition averaged over DBH genotypes and for individuals with CC and CT/TT genotypes separately are shown in Fig. 1.

### Functional connectivity

**Fig. 2:**
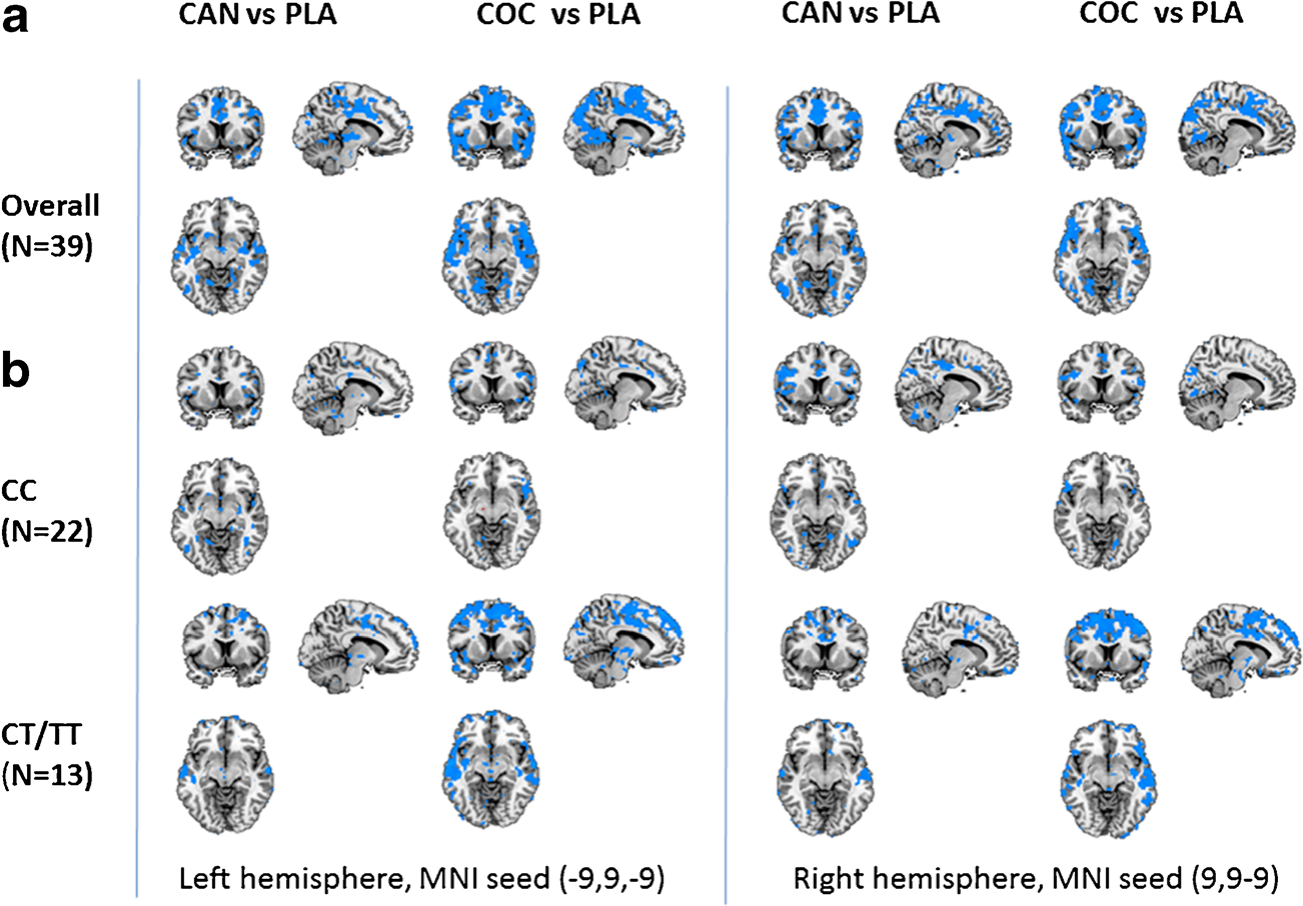
NAc related functional connectivity decrements following cannabis and cocaine in the *left* and *right* hemisphere, relative to placebo. Shown are thresholded (*t* = 2.34) Z-score maps of functional connectivity (**a**) averaged over DBH genotypes and (**b**) for individuals with CC and CT/TT genotypes separately (CAN = cannabis, COC = cocaine; left = left; planes are made at MNI seed position)

GLM contrast showed that overall, cannabis as well as cocaine significantly reduced functional connectivity with NAc seeds in both hemispheres, relative to placebo. Reductions in functional connectivity were prominent in the cingulate gyrus as well as in broad areas of the frontal, temporal, parietal and occipital lobes. GLM contrasts did not reveal any positive changes (drug > placebo) in functional connectivity. Figure 2 shows the decrements in functional connectivity for each drug-placebo contrast across DBH genotypes and for individuals with CC and CT/TT genotypes separately.

**Fig. 3:**
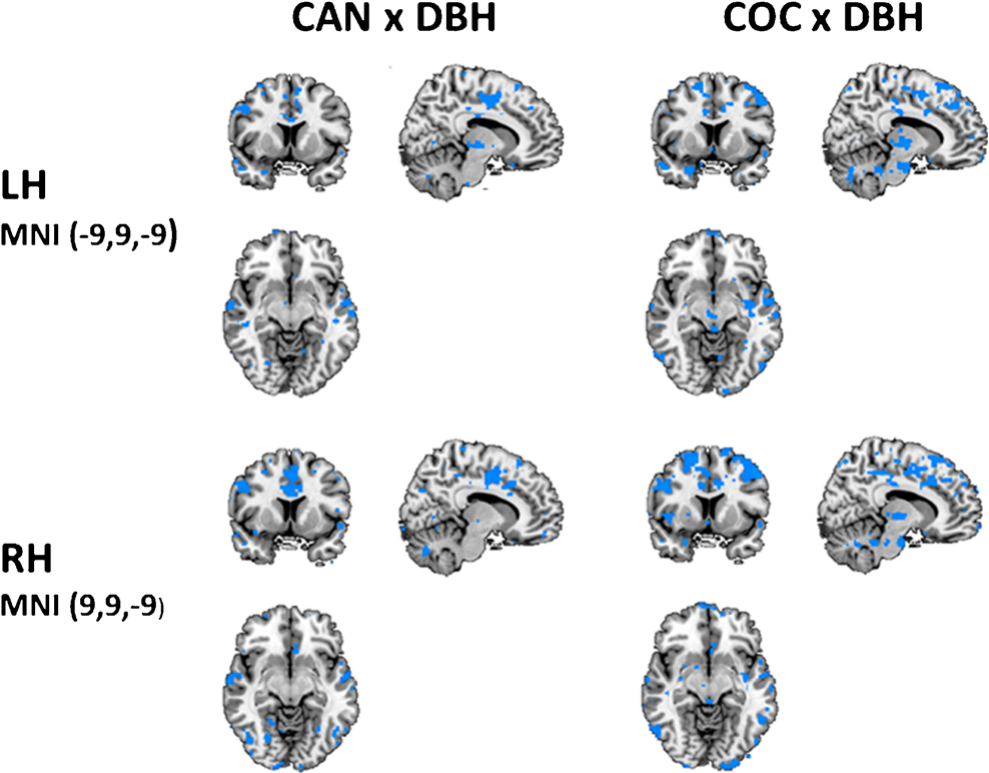
NAc related functional connectivity decrements as a function of cannabis x DBH and cocaine x DBH interactions in the *left* (LH) and *right* hemisphere (RH), (CAN = cannabis, COC = cocaine; left = left; planes are made at MNI seed position)

GLM analyses of interaction between DBH genotype and cannabis and cocaine revealed that reductions in functional connectivity in striatum, thalamus, cingulate gyrus and frontal, parietal and temporal lobes were most prominent in individuals with CT/TT genotypes. There was no main effect of DBH genotype. Figure 3 shows significant interactions between drug-placebo contrast and DBH genotype for NAc seeds in both hemispheres. A summary of brain areas showing significant decrements in functional connectivity with the NAc is given in Table 1. Reported are data obtained from the NAc seed in the right hemisphere. These were generally identical to those obtained for the NAc seed in the left hemisphere (not shown).

**Table 1:** Summary of brain areas showing decrements in functional connectivity with the NAc (right hemisphere) following cannabis and cocaine and their interaction with DBH genotype

Overall	BA	Voxel	x	y	z	FWE
Cannabis vs placebo						
Left, frontal, limbic lobe, (anterior) cingulate gyrus	6,9, 24, 32	24,785	−36	−4	42	.000
Left frontal lobe, Right frontal lobe, Left thalamus	47	373	−18	−32	−8	.002
Left, temporal lobe, cerebellum	36	374	−48	−36	−26	.000
Left, temporal lobe, occipital lobe	37,38	1213	−32	−48	−16	.000
Right, temporal lobe, middle temporal gyrus	21	741	66	−8	−10	.000
Right. Occipital lobe, cerebellum	18	313	30	−78	−8	.003
Right, insula, claustrum	13	430	43	4	1	.003
Cocaine vs placebo						
Left, parietal lobe, temporal lobe	5,7,22, 39, 40	30,270	−38	−44	66	.000
Left, occipital lobe, Right cerebellum	18	903	−20	−64	−8	.000
Right, left frontal, limbic lobe, (anterior) cingulate gyrus	9, 24, 32	1021	40	26	36	.000
Cannabis x DBH						
Left, frontal and parietal lobe	3,6	474	−36	−4	42	.000
Left, parietal lobe	5,7,40	397	−28	−50	62	.000
Right, temporal and parietal lobe	19,39	512	32	−74	30	.000
Right, limbic lobe, (anterior) cingulate gyrus	24,32	12,000	4	−4	33	.000
Cocaine x DBH						
Left and right frontal lobe; Right, limbic lobe, (anterior) cingulate gyrus	8,9, 24, 32	10,580	−8	40	53	.000
Left, parietal lobe	40	19,330	−58	−40	38	.000
Left, temporal lobe, putamen, globus pallidus, thalamus	21,22	22,670	−21	−6	11	.000
Right, parietal and temporal lobe	22,40	13,920	50	−40	34	.000
Right, parietal, temporal, occipital lobe	19,39	10,090	26	−66	−28	.000
Right, cerebellum, Limbic lobe, parahippocampal gyrus	28,34,36	13,260	26	−66	−28	.000

### Correlation of MFFT and functional connectivity data

**Fig. 4:**
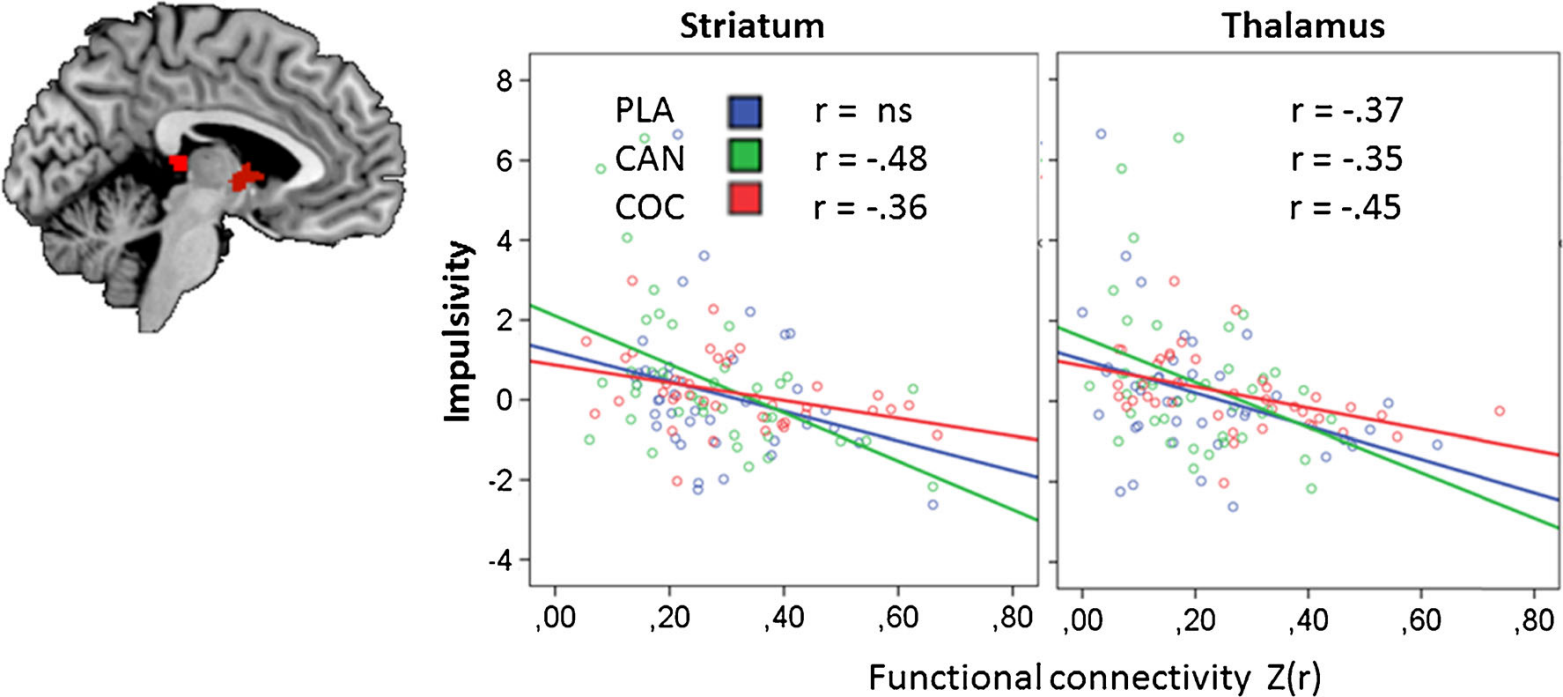
Significant correlations in the striatum and the thalamus between functional connectivity and the MFFT measure of impulsivity in each treatment condition (CAN = cannabis, COC = cocaine, PLA = placebo), across DBH genotypes

Voxel wise correlation analysis revealed significant (negative) correlations between Impulsivity scores and functional connectivity in the striatum (MNI peak: -2, 2, 4) and thalamus (MNI peaks: -2, -28, 10; 4,-20, 16) across genotypes, indicating that Impulsivity levels increased when functional connectivity decreased. Correlations between functional connectivity and the MFFT measure of Impulsivity in the striatum and the thalamus were generally significant in all treatment conditions as shown in Fig. 4. Overall, correlation between Impulsivity and functional connectivity were comparable for CC individuals (*r* = −.38 and −.33) and CT/TT individuals (*r* = −.27 and −.35) across treatments in the 2 identified areas. Efficiency was not significantly correlated with functional connectivity data.

### Control measures

One-way ANOVA revealed that DBH groups did not differ in cannabis and cocaine use over the previous 3 months. Likewise, age, gender distribution and trait impulsivity (as assessed with the Barratt Impulsiveness Scale) did not differ between groups. Finally, THC and cocaine concentrations did also not significantly differ between DBH groups. Mean concentrations of THC, cocaine and their main metabolites averaged over groups are given in Table 2.

**Table 2:** Mean (sd) concentrations of THC, cocaine and their main metabolites at baseline (T0) and prior to MFFT (T1) and resting state fMRI (T2)

	THC [ng/ml]	THC-OH [ng/ml]	THC-COOH [ng/ml]	Cocaine [mg/L]	BZE [mg/L]	EME [mg/L]
*T0*	1.57 (4.14)	0.62 (1.72)	20.91 (37.68)	0.00 (0.0)	0.00 (0.00)	0.00 (0.00)
*T1*	73.81 (63.09)	6.86 (4.32)	38.69 (33.38)	0.25 (0.19)	0.49 (0.27)	0.14 (0.11)
*T2*	46.00 (34.19)	5.79 (3.51)	42.27 (36.03)	0.31 (0.18)	1.12 (0.37)	0.26 (0.10)

## Discussion

MFFT data indicated that both cannabis and cocaine significantly increased cognitive impulsivity in individuals with CT/TT genotypes but not in CC homozygotes. The former changed from a reflective decision style during placebo (i.e. low impulsivity scores) to an impulsive decision style during drug intoxication (i.e. higher impulsivity scores). This is illustrated by the finding that CT/TT participants responded more rapidly at the cost of making more errors during cocaine and made more errors despite prolonged decision times during cannabis intoxication. CC homozygotes revealed relatively high impulsivity scores during placebo that did not alter during drug intoxication. Single doses of cannabis (Ramaekers et al. 2006a) and cocaine (van Wel et al. 2013) have previously been reported to increase motor and cognitive impulsivity, particularly at higher doses (Fillmore et al. 2006) or higher drug concentrations (Ramaekers et al. 2009). Based on the present study it now appears that changes in impulsive decision making may be limited to individuals with CT/TT genotypes and that their (default) reflective operation mode is more prone to the disinhibiting effect of cannabis and cocaine.

Both drugs also decreased synchronicity between BOLD responses in the NAc and other areas of the brain. In particular, cannabis and cocaine decreased functional connectivity between the NAc and the limbic lobe (i.e. anterior cingulate), medial prefrontal cortex, striatum and thalamus. Reductions in functional connectivity were most prominent in individuals with CT/TT genotypes. These decrements may be largely related to the moderating effects of cannabis and cocaine on GABAergic projections to output nuclei within the basal ganglia. Both drugs elevate dopamine levels in the NAc (Bossong et al. 2009; Kalivas and Duffy 1990; Weiss et al. 1992) which in turn increases inhibitory GABAergic neurotransmission to the ventral pallidum through D1 receptor stimulation in the direct pathway. Inhibitory output to the ventral pallidum leads to reduction of GABAergic, inhibitory tone to the dorsomedial nucleus of the thalamus, which, in turn, projects to the limbic areas of the cerebral cortex (Kalivas and Volkow 2005; Nakanishi et al. 2014; Pierce and Kumaresan 2006). In addition, dopamine also turns down the inhibitory, indirect striatal pathway from the NAc to the thalamus via D2 receptor activation (Nakanishi et al. 2014). The net effect of dopaminergic stimulation of D1 and D2 receptors in the NAc is therefore ‘disinhibition’ and increased thalamic feedback on pleasure and reward to the cerebral cortex. Loss of functional connectivity may reflect elevation of striatal ‘bottom-up’ impulses at the cost of ‘reflective’ top-down control from frontal parts in the limbic circuit. Indeed, correlational analyses confirm a strong association between cognitive impulsivity and functional connectivity in the subcortical areas (i.e. striatum, thalamus) of the brain. Impulsivity increased with lower functional connectivity in these areas, and this association was strongest in the drug conditions. This corresponds with the notion of striatal overdrive due to increased dopaminergic stimulation of the NAc and the subsequent ‘disinhibition’ of the thalamus which acts as the major relay between the reactive and reflective part of the reward system.

Drug induced disinhibition of corticostriatal circuits and subsequent loss of cognitive control may also provide an important mechanism by which drug users can progress to drug seeking. In the present study, loss of functional corticostriatal connectivity was particularly prominent in the (supracallosal) anterior cingulate and medial prefrontal cortex. These areas are implicated in detection of salient or erroneous events and the signaling of top down reorientation and implementation of attention (Beckmann et al. 2009; Sutherland et al. 2012). Reduced brain activity in these areas has been associated with comprised abilities to exert control over strong prepotent urges in cocaine users (Hester and Garavan 2004). Reductions in functional corticostriatal connectivity have also been reported in chronic users of cocaine (Camchong et al. 2014; Gu et al. 2010; Tomasi et al. 2010) and cannabis (Fischer et al. 2014) during abstinence. This suggests that a drug induced imbalance in the corticostriatal circuitry can endure during abstinence in chronic users and lead to long lasting disinhibition.

Several potential limitations of the current study should be addressed. First, sample size in the current study, appears low to study genetic polymorphism. It has been argued that sample size should be well above a hundred participants or more to reliable assess genetic –environment (e.g. drug) interactions (Duncan and Keller 2011). The implication of a low sample size is that true genotype × drug effects may go undetected or that rate of false discoveries progressively increases with the number of candidate genes that are being screened for drug interactions (Duncan and Keller 2011). This argument is particularly true for association studies that randomly screen for a multitude of candidate gene-drug interactions. However, the present experimental study instead tested a single hypothesis about a single candidate gene (i.e. DBH) to exclude risk of chance capitalization and type 1 statistical errors. In such a scenario, sample size considerations are essentially not different from those in any other study making between group comparisons. The current study was well powered to demonstrate drug x DBH interactions in the MFFT. Sample size for resting state data was lower, but still within a sufficient range to pick up drug effects as demonstrated in previous studies (Honey et al. 2003; Kelly et al. 2009; Ramaekers et al. 2013; Schrantee et al. 2015). Moreover, pearson r correlations between functional connectivity and MFFT demonstrated a significant association between these 2 datasets, with coefficients varying between *r* = −30 and *r* = −50 indicating medium to large effect sizes. We therefore believe that the current DBH x drug interaction on functional connectivity is a true finding that is in support of the same interaction that we observed in the cognitive impulsivity data set in a much larger sample size.

Second, several study participants tested positive for THC at baseline prior to drug and placebo administration. However, mean baseline THC concentration was very low and below the threshold (i.e. < 2 ng/mL) above which performance impairment at the group level becomes apparent (Ramaekers et al. 2006b). Moreover, THC concentrations did not differ between DBH groups at baseline and during treatments. Therefore differential cannabis effects on MFFT and functional connectivity observed between DBH groups cannot be explained by differential THC concentration but must be caused by a factor that affects sensitivity to THC, in this case DBH genotype.

Cannabis and cocaine are also well known to affect cardiovascular variables. Cannabis decreases blood pressure shortly after smoking and increases heart rate (Ramaekers et al. 2009) whereas cocaine causes increments in both of these parameters during intoxication (Fillmore et al. 2002). In the current study we did not measure heart rate and blood pressure during resting state to control for cardiovascular influences on the BOLD response. Instead we removed ‘noise’ attributable to such physiological parameters by low band-pass filtering (0.01–0.08 Hz) of the preprocessed data. Moreover, the potential of drug induced cardiovascular influences on the BOLD response is similar across DBH groups and therefore cannot explain drug x DBH interactions as observed in the present study.

The present study included an unequal distribution of males and females. In total, 122 regular users of cannabis and cocaine entered the study of which 26 were females. The gender distribution did not differ between DBH groups. Sample sizes of males and females did not allow for further gender specific analyses, particularly of the resting state dataset. Further studies however may wish to address the issue of gender specificity in terms of drug x DBH interactions since several studies have demonstrated gender specific responses to reward related decision processing (Diekhof et al. 2012; Lighthall et al. 2012).

The present study demonstrated that individual responses to cannabis and cocaine challenges vary with DBH genotype. Heterogeneity in response to drug challenges may also have important clinical implications as these might be useful for developing personalized, targeted treatment in subgroups of drug dependent patients. Increased sensitivity to dopaminergic challenges make individuals with CT/TT genotypes more prone to downregulation and depletion of the dopaminergic system as has been observed in chronic drug users (Bough et al. 2014). Preliminary data suggests that replenishment of dopamine through pharmacotherapy would therefore be more successful in patients with CT/TT genotypes as compared to patients with CC genotypes (Liu et al. 2014).
